# Evaluation of the genetic relationship between high elevation pulmonary arterial pressure with moderate elevation with feedlot and carcass performance

**DOI:** 10.1093/tas/txaa113

**Published:** 2020-12-22

**Authors:** Emma A Briggs, Richard Mark Enns, Milton G Thomas, Tim N Holt, Scott E Speidel

**Affiliations:** 1 Department of Animal Science, Colorado State University, Fort Collins, CO; 2 Department of Clinical Sciences, Colorado State University, Fort Collins, CO

## INTRODUCTION

In areas of high elevation, hypoxia-induced pulmonary hypertension (PH) causes right ventricle failure and can lead to manifestation of high mountain disease, often called brisket disease. The limited amount of oxygen available in areas of high elevation causes vasoconstriction in pulmonary vasculature (Farber and Loscalzo, 2004). Holt and Callan (2007) reported that approximately 5% death loss for cattle who reside in high elevation regions could be attributed to PH. Fortunately, pulmonary arterial pressure (PAP) can be used as an indicator trait which identifies cattle who have the potential to develop (PH). Neary et al. (2015) suggested that as cattle enter feedlot systems, an increase in adipose tissue and ruminal engorgement could increase PAP. Rapid weight gain and high intake of concentrate diets for cattle in feedlots cause increased work of cardiac ventricles and sustained accelerated circulation (Jensen et al., 1976).

Feed efficiency, a ratio of inputs to outputs, is important in the beef industry as feed costs represent up to 70% of total expenses of production (Shike, 2013). Also, premiums for higher quality carcasses, as well as an increase in popularity for branded beef programs has drawn interest in the genetic merit of carcass traits (CT). As indexes of expected progeny difference (EPD) are becoming commercially available for these traits, its logical that enhanced selection pressure is occurring in breeding objectives. Also, since American Angus Association is publishing a PAP EPD, it is crucial to understand the potential antagonisms between PAP and feedlot performance and carcass quality. We hypothesized that cattle with higher yearling PAP scores at high elevation would show a general decrease in feedlot performance and an increase in fat-related CT when finished at moderate elevations. Therefore, the objective of this study was to determine if a genetic relationship exists between PAP and feedlot performance and CT.

## MATERIALS AND METHODS

The Institutional Animal Care and Use Committee and Colorado State University (approval number 17-7179A) approved all animal procedures.

### Animal Management and Data Collection

Cattle used for this study were from Colorado State University Beef Improvement Center (CSU-BIC; elevation 2,115 m) Angus spring calving herd. Pulmonary arterial pressure measurements were collected from 6,898 animals at the CSU-BIC from the years 1993 to 2017 with an average test age of 339 d.

After weaning, steer calves were moved and placed in the Colorado State University Feed Intake Unit (1,557 m) to collect individual feed intake measurements using the Growsafe Monitoring Systems. Before entering the intake test, cattle were processed and sorted upon arrival and placed into group pens based on starting weight. Start age and length of test varied by year and are further described in Table 1. All steers had a 21 d warm-up period to adapt to testing facilities and diet. Test diet was consistent through all 5 yr of testing and is further detailed in Table 2. Cattle were weighed every 2 wk to obtain average daily gain (ADG). After completion of the feed intake test, steers were moved and finished at the Eastern Colorado Research Center (1,420 m). Of the 1,627 cattle harvested for this study, feedlot performance data were collected on 558 steers.

**Table 1.** Feed intake test lengths and average starting age

Test year	Length of test in days	Number of steers	Average starting age
2013	70	92	490
2014	77	110	455
2015	69	109	375
2016	74	96	376
2017	73	126	277

**Table 2.** Feed ration utilized for intake study

	Ration %
Corn silage	10%
Alfalfa hay	6.90%
Cracked corn	74.46%
Dry distillers grain	3.79%
Limestone	0.75%
Mineral supplement	4.10%

### Statistical Analysis

Heritabilities and genetic correlations were obtained using the software package ASReml 3.0 (Gilmour et al., 2009). For this study, 5-trait models were analyzed to estimate the genetic relationship between PAP, feedlot performance, and carcass quality. In each model, traits of PAP, ADG, average dry matter intake (ADMI), and weaning weight (WW) were included in the mixed model with a single CT alternating through the series. The CT traits included in this analysis were rib eye area (REA), marbling score (MARB), back fat (BF), hot carcass weight (HCW), and calculated yield grade (CYG).

Total pedigree size for each 5-trait analysis was 12,699, which consisted of 348 unique sires and 1,904 unique dams with an average inbreeding level of 0.016. In the series of models, the fixed effects for PAP included PAP contemporary group (CG; sex, PAP date, and PAP score) and PAP age as a linear covariate (LC). For feedlot performance traits, fixed effects included test length, feed intake test CG (weaning date and intake test pen), and starting age as a LC. Each CT included the fixed effects of feed intake test CG, slaughter date, and slaughter age as a LC. For PAP, feedlot, and CT, individual animal was the sole random effect which was included for the direct additive genetic effect. Fixed effects for WW included WW CG (weaning date), Beef Improvement Federation (BIF) adjusted age of dam, sex, and weaning age as a LC as well as direct additive, maternal, and maternal permanent environmental random effects (BIF, 2018).

The following is the general matrix form for the equation used for the analysis:

$$\begin{array}{ll} \left[\begin{array}{l} y_{1}\\ y_{2}\\ y_{3}\\ y_{4}\\ y_{5} \end{array}\right]= & \left[\begin{array}{ccccc} X_{1} & 0 & 0 & 0 & 0\\ 0 & X_{4} & 0 & 0 & 0\\ 0 & 0 & X_{3} & 0 & 0\\ 0 & 0 & 0 & X_{4} & 0\\ 0 & 0 & 0 & 0 & X_{5} \end{array}\right]\;\left[\begin{array}{l} b_{1}\\ b_{2}\\ b_{3}\\ b_{4}\\ b_{5} \end{array}\right]\;\\ & +\left[\begin{array}{ccccccc} Z_{a_{1}} & 0 & 0 & 0 & 0 & 0 & 0\\ 0 & Z_{a_{2}} & 0 & 0 & 0 & 0 & 0\\ 0 & 0 & Z_{a_{3}} & 0 & 0 & 0 & 0\\ 0 & 0 & 0 & Z_{a_{4}} & 0 & 0 & 0\\ 0 & 0 & 0 & 0 & Z_{a_{5}} & 0 & 0\\ 0 & 0 & 0 & 0 & 0 & Z_{m_{5}} & 0\\ 0\; & 0 & 0 & 0 & 0 & 0 & Z_{p_{5}} \end{array}\right]\;\left[\begin{array}{l} \begin{array}{l} u_{a_{1}}\\ u_{a_{2}} \end{array}\\ u_{a_{3}}\\ u_{a_{4}}\\ u_{a_{5}}\\ u_{m_{5}}\\ u_{p_{5}} \end{array}\right]+\left[\begin{array}{l} e_{1}\\ e_{2}\\ e_{3}\\ e_{4}\\ e_{5} \end{array}\right], \end{array}$$

where *y*_*i*_ is a vector of observations for the *i*th trait, *X*_*i*_ is an incidence matrix relating unknown fixed effects solutions in *b*_*i*_ to observations in *y*_*i*_, $Z_{a_{i}}$ is an incidence matrix relating unknown additive (*a*) random effects solutions in $u_{a_{i}}$ to observations in *y*_*i*_, $Z_{m_{5}}$ is an incidence matrix relating unknown maternal (*m*) random genetic effects solutions in $u_{m_{5}}$ to observations in *y*_*i*_, $Z_{p_{5}}$ is an incidence matrix relating unknown maternal permanent environmental (*p*) random additive effects solutions in $u_{p_{5}}$ to observations in *y*_*i*_, and *e*_*i*_ is a vector of random residual errors for each record.

With (co)variances equal to:

$$\begin{array}{l} Var\left[\begin{array}{l} u_{a1}\\ u_{a2}\\ u_{a3}\\ u_{a4}\\ u_{a5}\\ u_{m5} \end{array}\right]=\left[\begin{array}{cccccc} \sigma_{a1}^{2} & \sigma_{a1a2} & \sigma_{a1a3} & \sigma_{a1a4} & \sigma_{a1a5} & \sigma_{a1m5}\\ \sigma_{a2a1} & \sigma_{a2}^{2} & \sigma_{a2a3} & \sigma_{a2a4} & \sigma_{a2a5} & \sigma_{a2m5}\\ \sigma_{a3a1} & \sigma_{a3a2} & \sigma_{a3}^{2} & \sigma_{a3a4} & \sigma_{a3a5} & \sigma_{a3m5}\\ \sigma_{a4a1} & \sigma_{a4a3} & \sigma_{a3a4} & \sigma_{a4}^{2} & \sigma_{a4a5} & \sigma_{a4m5}\\ \sigma_{a5a1} & \sigma_{a5a2} & \sigma_{a5a3} & \sigma_{a5a4} & \sigma_{a5}^{2} & \sigma_{a5m5}\\ \sigma_{m5a1} & \sigma_{m5a2} & \sigma_{m5a3} & \sigma_{m5a4} & \sigma_{m5a5} & \sigma_{m5}^{2} \end{array}\right]⊗A \end{array}$$

maternal permanent environmental variance equal to:

$$\begin{array}{l} MPE=Var\left[u_{p_{5}}\right]I \end{array}$$

and residual (co)variance equal to:

$$\begin{array}{l} Var\left[\begin{array}{l} \begin{array}{l} e_{1}\\ e_{2} \end{array}\\ e_{3}\\ e_{4}\\ e_{5} \end{array}\right]=\left[\begin{array}{ccccc} \sigma_{e_{1}}^{2} & \sigma_{e_{12}} & \sigma_{e_{13}} & \sigma_{e_{14}} & \sigma_{e_{15}}\\ \sigma_{e_{21}} & \sigma_{e_{2}}^{2} & \sigma_{e_{23}} & \sigma_{e_{24}} & \sigma_{e_{25}}\\ \sigma_{e_{31}} & \sigma_{e_{32}} & \sigma_{e_{3}}^{2} & \sigma_{e_{34}} & \sigma_{e_{35}}\\ \sigma_{e_{41}} & \sigma_{e_{42}} & \sigma_{e_{43}} & \sigma_{e_{4}}^{2} & \sigma_{e_{45}}\\ \;\sigma_{e_{51}} & \sigma_{e_{52}} & \sigma_{e_{53}} & \sigma_{e_{54}} & \sigma_{e_{5}}^{2} \end{array}\right]⊗I \end{array}$$

where *A* is Wright’s numerator relationship matrix, $\sigma_{ai}^{2}$ is the direct genetic variance for trait *i*, $\sigma_{mi}^{2}$ is the maternal genetic variance for trait *i*, $\sigma_{aij}$ is the direct genetic covariance between trait *i* and *j*, $\sigma_{aimi}$ is the covariance between the direct component of trait *i* and the maternal component of trait *i*, $\sigma_{ei}^{2}$ is the residual variance for trait *i*, and $\sigma_{eij}$ is the residual covariance of traits *i* and *j*, ⊗ is the Kronecker product operator *I* was an identity matrix with an order equal to the number of observations in *y*_*i*_ (Wright, 1992).

## RESULTS AND DISCUSSION

Summary statistics for PAP, feedlot, and CT are detailed in Table 3. Results from this analysis included heritability estimates and genetic correlations and are detailed in Table 4. Average PAP heritability (0.29 ± 0.03) was consistent with previously reported literature estimates (0.26 ± 0.03 to 0.46 ± 0.16; Enns et al., 1992; Crawford et al., 2016). Feedlot performance estimates were moderately heritable, with average estimates being 0.31 ± 0.11 (ADG) and 0.28 ± 0.11 (ADMI). Estimates were similar with previously reported literature for both ADG (0.35 ± 0.03 to 0.41 ± 0.08; Arthur et al., 1997; Schenkel et al., 2004) and ADMI (0.39 ± 0.03 to 0.42 ± 0.13; Arthur et al., 2001; Elzo et al., 2009). Carcass traits (REA, MARB, HCW, and CYG) were moderately heritable with estimates of, 0.27 ± 0.05, 0.27 ± 0.06, 0.27 ± 0.06, and 0.44 ± 0.06, respectively.

**Table 3.** Summary statistics for traits included in analysis

	*N*	Mean	SD	Min	Max
PAP, mmHg	6,898	42.28	9.61	21	139
WW, kg	9,026	214.08	30.90	97.98	368.32
ADG, kg/d	558	1.656	0.286	0.30	2.44
ADMI, kg/d	558	11.49	2.33	4.34	19.20
REA, cm^2^	1,627	80.97	9.29	35.48	119.99
MARB	1,627	585.53	116.74	90.00	970.00
BF, mm	1,627	14.478	3.81	2.54	43.68
HCW, kg	1,627	382.99	46.93	171.46	519.36
CYG	1,499	3.55	0.56	1.50	5.00

PAP = Pulmonary arterial pressure, WW = Weaning weight, ADG = Average daily gain, ADMI = Average dry matter intake, REA = Rib eye area, MARB = Marbling score, BF = Back fat, HWC = Hot carcass weight, CYG = Calculated yield grade.

**Table 4.** Heritability estimates (diagonal) (SE) and genetic correlations (above diagonal) (SE)

	PAP	ADG	ADMI	REA	MARB	BF	HCW	CYG	WWD	WWM
PAP	0.29 (0.03)^*a*^	0.05 (0.17)^*a*^	0.34 (0.20)^*a*^	−0.30 (0.12)	0.00 (0.13)	−0.07 (0.13)	0.14 (0.10)	0.29 (0.13)	0.13 (0.10)^*a*^	0.07 (0.10)^*a*^
ADG		0.31 (0.11)^*a*^	0.46 (0.21)^*a*^	0.59 (0.17)	0.30 (0.20)	0.36 (0.19)	0.83 (0.09)	0.50 (0.18)	0.78 (0.14)^*a*^	0.12 (0.23)^*a*^
ADMI			0.28 (0.11)^*a*^	0.35 (0.23)	0.02 (0.26)	0.34 (0.22)	0.66 (0.14)	0.47 (0.22)	0.29 (0.22)^*a*^	0.58 (0.27)^*a*^
REA				0.27 (0.05)	—	—	—	—	0.21 (0.14)	0.10 (0.15)
MARB					0.27 (0.06)	—	—	—	0.20 (0.15)	−0.14 (0.16)
BF						0.27 (0.06)	—	—	0.03 (0.15)	0.31 (0.16)
HCW							0.44 (0.06)	—	0.50 (0.10)	0.54 (0.10)
CYG								0.25 (0.06)	0.23 (0.15)	0.53 (0.15)
WWD									0.16 (0.03)^*a*^	−0.20 (0.12)^*a*^
WWM										0.13 (0.03)^*a*^

PAP = Pulmonary arterial pressure, WW = Weaning weight, ADG = Average daily gain, ADMI = Average dry matter intake, REA = Rib eye area, MARB = Marbling score, BF = Back fat, HWC = Hot carcass weight, CYG = Calculated yield grade.

^*a*^Reported as the average estimate and largest SE of all 5-trait analyses.

Genetic correlations between PAP and feedlot performance traits were found to be low to moderately correlated with ADG and ADMI reported average being 0.05 and 0.34, respectively. Consistent with these results, Maddock et al. (2010) reported in a multibreed study, that growing beef cattle with lower PAP had decreased ADMI. Pauling (2017) reported in a study of Angus cattle that high PAP may be slightly associated with increasing growth and muscle mass. Also, a study following a group of Angus steers from a high elevation operation to a moderate elevation feedlot, concluded that while in the feedlot, fatter and larger framed cattle appeared to have high higher PAP (Neary et al., 2015). Genetic correlations between PAP and weaning weight direct (WWD) and weaning weight maternal (WWM) were low but positive, with an average of 0.10 and 0.09, respectively. Results for WWD are slightly lower than other reported values on similar cattle with estimates of 0.14 ± 0.15 to 0.20 ± 0.04 (Crawford et al., 2016; Pauling, 2017). Genetic relationship between PAP with REA, MARB, BF, HCW, and CYG was −0.30 ± 0.12, 0.00 ± 0.13, −0.07 ± 0.13, 0.14 ± 0.10, and 0.29 ± 0.13 respectively. Pauling (2017) described similar results from a study analyzing the genetic relationship between PAP with carcass ultrasound measurements of REA, intramuscular fat and BF to be 0.24 ± 0.12, −0.04 ± 0.10, and −0.03 ± 0.12, respectively. These results suggest that selection against high PAP animals will not drive disadvantageous influence on CT.

In summary, high elevation cattle with high PAP could suffer from poor cardiopulmonary health, feed efficiency, and carcass quality at moderate elevation feedlots. Genetic correlation between PAP with CYG, REA, BF, and HCW suggests that cattle with lower PAP could result in a heavy muscled, leaner carcass when compared with high PAP cattle. Additionally, the more substantial genetic correlation between PAP and ADMI suggests that higher PAP cattle are less efficient at converting feed. High PAP cattle entering the feedlot at moderate elevations could be using excess energy to their pulmonary cardiovascular system resulting in a less feed efficient animal with marginal carcass quality.

## IMPLICATIONS

Results from this study suggest that selection against high PAP will not negatively influence feedlot performance and carcass quality for subsequent generations. Unfortunately, cattle culled from high elevation herds due to high PAP could have a reduction in feed efficiency compared with their contemporaries when relocated to a moderate elevation feedlot for finishing. With the rising cost of finishing cattle, these findings advocate that feedlots can purchase cattle from producers with selection pressure on PAP in their breeding objective without subsequently having undesirable feedlot and CT.



*Conflict of interest statement*. None declared.
